# The influence of restoration measures on phosphorus internal loading from the sediments of a hypereutrophic lake

**DOI:** 10.1007/s11356-017-8997-2

**Published:** 2017-04-22

**Authors:** Katarzyna Kowalczewska-Madura, Renata Dondajewska, Ryszard Gołdyn, Stanisław Podsiadłowski

**Affiliations:** 1 Faculty of Biology, Department of Water Protection, Adam Mickiewicz University, Umultowska 89, 61-614 Poznań, Poland; 2 Institute of Biosystems Engineering, University of Life Sciences, Wojska Polskiego 50, 60-627 Poznań, Poland

**Keywords:** Hypereutrophic lake, Sustainable restoration, Phosphorus release, Bottom sediments, Iron treatment, Nitrate application

## Abstract

Uzarzewskie Lake was a hypereutrophic, dimictic lake characterized by low water transparency, high chlorophyll-a concentration and intense phytoplankton blooms; thus, restoration treatment was started. A chemical treatment, based on phosphorus inactivation with small doses of iron sulphate, was undertaken in 2006–2007. Nitrate-rich groundwater flowing from seepage springs was directed into the lake hypolimnion to increase redox potential since 2008. Phosphorus internal loading was reduced by more than 80% as a result of restoration treatment. In the profundal zone, where P release from the bottom sediments prevailed throughout the year, it decreased from 13.02 mg P m^−2^ day^−1^ in 2010 to 2.81 mg P m^−2^ day^−1^ in 2015. Meanwhile, in the littoral zone, P accumulation in bottom sediments predominated; hence, the mean value of internal loading was increasing from 2.61 mg P m^−2^ day^−1^ in 2011 to 10.24 mg P m^−2^ day^−1^ in 2015. The annual P load from the bottom sediments in the profundal zone was much higher than from the littoral zone as a result of (i) a higher P release in most years and (ii) the greater area of this zone (82% of the lake’s area). The fraction of residual phosphorus (Res-P) has the largest share, and in recent years, this has shown a tendency to decrease. The sum of bioavailable fractions was low (average 5.7%) showing a downward trend in recent years. Sustainable restoration of the lake resulted in a slow but steady decreasing trend in internal phosphorus loading.

## Introduction

Biogenic compounds, phosphorus in particular, are considered to be the most important factor influencing lake water quality. Phosphorus accumulated in sediments during periods of significant external loading is subsequently very often released from sediments to the water column, and thus, sediments become a source of internal load, maintaining high trophic state and bad water quality (Søndergaard et al. 2001; Malmaeus and Rydin 2006). Transport of the element or its compounds from sediments to the water, prevailing sedimentation from the water to sediments, is defined as “release from sediments” or “internal loading” (Boström et al. 1988). Therefore, bottom sediments are an important part of the ecosystem and their main significance is connected with their ability to store and release phosphorus. They may serve as a trap or a source of phosphorus for the water column, especially in the state of hypereutrophy (Boström et al. 1988; Wang et al. 2009; Kowalczewska-Madura et al. 2010). Generally, phosphorus internal loading from bottom sediments is the resultant between “the downward flow” caused by sedimentation of the particles constantly produced in the water column (algae, detritus) and “the upward flow” of phosphorus released as a result of organic matter mineralization, phosphorus gradient and transport mechanisms in the sediments (Søndergaard et al. 2002).

**Fig. 1:**
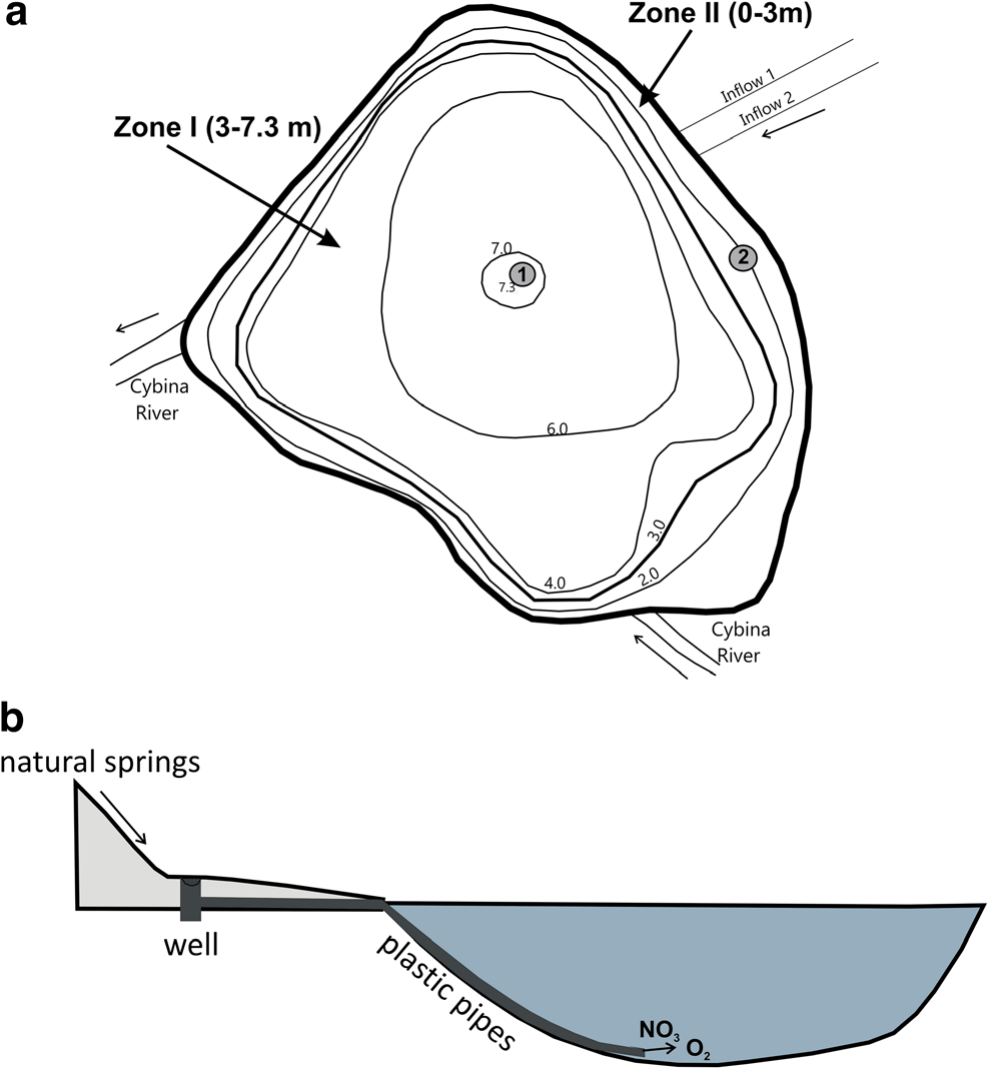
Location of sampling stations (*1* and *2*), the range of two zones in Uzarzewskie Lake and two inflows from the sources (according to Kowalczewska-Madura et al. 2015, changed) (**a**) and scheme of the system supplying hypolimnion zone with water from the sources (**b**)

Phosphorus release is believed to play a significant role in the process of eutrophication of water bodies (Gao et al. 2005; Brzozowska et al. 2013). Much attention was paid to reducing external loading of nutrients in order to improve water quality; however, internal loading from sediments, which was largely neglected, is also an important source for water eutrophication (Qin et al. 2016). The intensity and duration of P release may have a significant impact on its concentration in lake water and subsequently on lake water quality (Jeppesen et al. 1991; Gonsiorczyk et al. 1997). Lakes rich in nutrients with intense water blooms need restoration treatments to inactivate phosphorus in the bottom sediments (Søndergaard et al. 2002). Lake restoration also frequently requires decreasing P content in the water column through P adsorption and precipitation to the sediments (P inactivation) (Cooke et al. 1993; Kleeberg et al. 2013). In the case of Uzarzewskie Lake, iron treatment was first used in 2006–2007, namely as a solution of iron sulphate (commercial name PIX-112, containing 12% of Fe) in small doses 5.7–6.6 kg ha^−1^ (Kowalczewska-Madura et al. 2015). The objectives of this treatment were to precipitate phosphorus from the water column and increase the phosphorus-binding capacity of the sediments. As a result, internal loading was temporarily decreased, but water quality has improved only slightly. This was due to a deoxygenated meta- and hypolimnion with presence of hydrogen sulphide, indicating a very low redox potential. To increase this potential, a new sustainable method of restoration since 2008 was used (Gołdyn et al. 2014). Groundwater flowing from seepage springs, which was rich in nitrates and fully oxygenated, was directed into the lake hypolimnion to increase redox potential and to improve phosphorus binding with iron compounds. Water from a spring enters a special well from which it flows through a plastic pipe to the deepest part of the hypolimnion (Fig. 1a, b). The use of nitrates in lake restoration dates back to the 1970s, but until now, they were predominantly applied in one high dose (Ripl 1976). In Lake Uzarzewskie, nitrates are supplied to the lake bottom continuously in small amounts, which is an innovative approach, characteristic of sustainable restoration (Gołdyn et al. 2014).

The main objectives of the present study were to determine and compare the influence of sustainable lake restoration with the use of two different methods on the intensity as well as seasonal and spatial changes of phosphorus internal loading from the bottom sediments of hypereutrophic Uzarzewskie Lake.

## Materials and methods

Uzarzewskie Lake is a small, postglacial kettle-shaped water body, located at the 17th km of the course of the River Cybina, a right tributary of the River Warta (52° 27′ N, 17° 08′ E) (Western Poland). Although it is shallow (Table 1), it is a dimictic and bradymictic lake with short mixing periods. Uzarzewskie Lake is characterized by a thick layer of bottom sediments, which reaches several meters in its central part. They supplied the lake with a high internal load of nutrients (Kowalczewska-Madura et al. 2008, 2010).

**Table 1:** Morphometric data of Uzarzewskie Lake

Parameter	Unit	Value
Total area	ha	14.78
Area of open water	ha	10.6
Volume	m^3^	360,400
Mean depth	m	3.4
Maximum depth	m	7.3
Maximum length	m	450
Maximum width	m	330
Total catchment surface	km^2^	160.8
Direct catchment surface	km^2^	2.25

Source: Jańczak (1996) and Kowalczewska-Madura et al. (2015)

The waters of the River Cybina also supplied the lake with nutrients from the catchment area, i.e. mainly from farmlands and fish ponds. Wastewater, which was discharged to the lake from the tertiary SBR wastewater treatment plant in Uzarzewo village (until spring 2015), was also an important nutrient source. Research in the years 2005–2007 showed that in summer, the lake was characterized by oxygen depletion in the deeper layers of water and by high concentrations of total phosphorus and total nitrogen, reaching up to 2.33 mg P l^−1^ and 18 mg N l^−1^ (Kowalczewska-Madura et al. 2010). Water transparency decreased to 0.5 m and chlorophyll-a concentration increased up to 200 μg l^−1^ due to intense phytoplankton blooms, making recreational use impossible (Gołdyn et al. 2008; Budzyńska et al. 2009; Kozak 2009, Kozak and Gołdyn 2014). Therefore, it was decided to initiate restoration treatments. A chemical method was used in 2006–2007, based on a precise phosphorus inactivation with the use of iron sulphate (PIX-112) (Kowalczewska-Madura et al. 2015). PIX was dosed six times in 2006 and three times in 2007 in an amount ranging from 60 to 70 kg (380 and 180 kg in total, respectively), but the state of the lake remained hypereutrophic.

Another method was introduced in 2008 when two watercourses flowing from the springs at the bottom of the river valley slope were directed towards the hypolimnion in order to improve water oxygenation and to raise the redox potential of the bottom sediments. This spring water was characterized by a high concentration of nitrates (on average 37 mg N l^−1^), rather low temperature, which did not exceed 13.2 °C and low concentration of total phosphorus (mean value less than 0.07 mg P l^−1^) (Dondajewska et al. 2013).

Samples of bottom sediments were taken eight times in the period from February 2010 to November 2011 (once in each season) and 11 times from February 2013 to November 2015 at two stations (Fig. 1a). Station 1 was situated in the central part of the lake in the deepest place (7.3 m), whilst station 2 was in the littoral zone at a depth of 2 m. The ability of bottom sediments to release or accumulate P was determined on the basis of ex situ experiments with the use of intact sediment cores sampled with a modified Kajak sampler. Each transparent tube, 6 cm in diameter, containing the collected sample of intact sediment core (ca. 15 cm in length) and the overlying water, was sealed with rubber stoppers. Every sample was collected in three replicates. The cores were incubated under laboratory conditions at temperature and oxygen concentrations that corresponded to the ambient values determined during field research. Samples of water from above the sediment cores were collected at definite intervals (1–3 days) over a period of 2 weeks from every tube. Analyses of total phosphorus content were done spectrophotometrically with ascorbic acid as the reducer (ISO 2004). Water temperature, dissolved oxygen concentration, pH and conductivity were measured before water sampling in the tubes with a WTW Multi 350i meter. The data of studied parameters of the water overlying the cores of sediments is presented in Tables 2 and 3.

**Table 2:** The mean values (±SD) of physical parameters (mean of three replicates) in water overlying the sediment cores (the average of the experiment) sampled in Uzarzewskie Lake at station 1

Year	Season	Temperature (°C)	Oxygen (mg O_2_ l^−1^)	pH	Conductivity (μS cm^−1^)	ORP (mV)
2010	Winter	4.9 ± 0.16	0.81 ± 0.34	7.05–7.21	1035 ± 5.84	n.a.
	Spring	4.0 ± 0.53	0.26 ± 0.27	7.03–7.25	1093 ± 7.89	n.a.
	Summer	4.5 ± 0.0	0.31 ± 0.21	7.27–7.48	1070 ± 14.14	n.a.
	Autumn	4.4 ± 0.28	2.25 ± 0.82	7.34–7.89	979 ± 10.90	n.a.
2011	Winter	4.5 ± 0.23	5.52 ± 0.65	7.22–7.82	1158 ± 11.34	+163.0 to +147.0
	Spring	4.5 ± 0.24	4.84 ± 1.34	7.02–7.87	1078 ± 11.21	+142.0 to +116.2
	Summer	4.5 ± 0	0.06 ± 0.01	7.09–7.18	1002 ± 4.27	n.a.
	Autumn	4.3 ± 0.17	5.46 ± 2.79	7.54–7.96	787 ± 19.41	+71.0 to +66.5
2013	Winter	4.02 ± 0.26	8.15 ± 1.82	7.31–7.97	1013 ± 5.44	+167.4 to +144.5
	Spring	4.54 ± 0.82	1.13 ± 0.80	7.49–7.66	998 ± 11.50	n.a.
	Summer	4.6 ± 0.72	0 ± 0	7.15–7.40	1030 ± 4.35	n.a.
	Autumn	n.a.	n.a.	n.a.	n.a.	n.a.
2014	Winter	3.82 ± 1.21	4.38 ± 0.50	7.89–8.21	1012 ± 20.25	+140.8 to +194.1
	Spring	3.84 ± 0.23	4.23 ± 1.33	7.09–7.65	921 ± 41.42	+120 to +89.0
	Summer	4.9 ± 0.50	0.33 ± 0.34	7.43–7.71	963 ± 10.21	+116 to +107.8
	Autumn	3.75 ± 0.37	0.46 ± 0.38	6.95–7.02	803 ± 15.95	+146.0 to +166.4
2015	Winter	4.31 ± 0.75	1.74 ± 1.74	7.01–7.38	885 ± 25.30	+152.8 to +185.41
	Spring	4.3 ± 0.44	1.28 ± 1.28	7.96–8.02	841 ± 18.10	+132.6 to +141.9
	Summer	4.9 ± 0.25	0.24 ± 0.29	7.32–7.67	932 ± 7.14	+116.6 to -27.2
	Autumn	4.64 ± 0.34	0.98 ± 1.17	7.38–7.58	857 ± 24.3	+689 to−37.7

*n.a.* not analysed

**Table 3:** The mean values (±SD) of physical parameters (mean of three replicates) in water overlying the sediment cores (the average of the experiment) sampled in Uzarzewskie Lake at station 2

Year	Season	Temperature (°C)	Oxygen (mg O_2_ l^−1^)	pH	Conductivity (μS cm^−1^)	ORP (mV)
2010	Winter	4.9 ± 0.16	5.55 ± 1.4	7.20–8.04	1046 ± 13.3	n.a.
	Spring	5.0 ± 0.53	6.9 ± 0.5	7.66–8.05	922 ± 11.7	n.a.
	Summer	22.4 ± 1.83	0.24 ± 0.02	7.14–7.26	774 ± 25.5	n.a.
	Autumn	4.4 ± 0.28	7.50 ± 1.9	7.72–8.02	917 ± 11.1	n.a.
2011	Winter	4.4 ± 0.23	7.22 ± 0.64	7.23–7.73	1017 ± 9.75	+162.0 to +124.0
	Spring	4.5 ± 0.24	6.68 ± 0.46	7.30–8.0	854 ± 9.20	+115.5 to +138.0
	Summer	21.1 ± 0.64	4.40 ± 0.59	7.44–8.01	708 ± 21.6	n.a.
	Autumn	4.3 ± 0.17	10.23 ± 3.65	7.6–8.0	766 ± 10.1	+88.0 to +81.3
2013	Winter	3.96 ± 0.19	11.96 ± 3.19	7.86–8.10	943 ± 13.5	+165.7 to +144.9
	Spring	4.56 ± 0.79	7.76 ± 0.42	7.76–8.06	896 ± 8.6	n.a.
	Summer	16.93 ± 0.57	2.96 ± 0.59	7.90–8.35	799 ± 20.2	n.a.
	Autumn	n.a.	n.a.	n.a.	n.a.	n.a.
2014	Winter	3.82 ± 1.21	6.21 ± 0.86	8.28–8.56	987 ± 7.62	+130.8 to +103.7
	Spring	3.84 ± 0.23	5.27 ± 1.09	7.15–7.98	801 ± 55.19	+225.2 to +257.0
	Summer	17.85 ± 0.69	1.59 ± 0.97	7.48–7.85	741 ± 23.14	+102.2 to +65.5
	Autumn	3.75 ± 0.37	1.52 ± 0.23	7.05–7. 12	696 ± 13.41	+167.4 to +178.3
2015	Winter	4.31 ± 0.75	1.66 ± 0.61	7.04–7.45	872 ± 11.94	+116 to +154.0
	Spring	4.9 ± 0.25	2.75 ± 0.93	8.01–8.23	789 ± 36.6	+119.5 to +156.6
	Summer	18.3 ± 0.26	0.99 ± 0.29	7.53–7.98	720 ± 15.8	+99.0 to +109.0
	Autumn	4.64 ± 0.34	2.16 ± 1.71	7.66–7.80	684 ± 4.83	+77.8 to +116.6

*n.a.* not analysed

Ex situ experiments enabled us to determine whether the resultant process was the release or accumulation of phosphorus in the bottom sediments. Two zones differing in rates of P release from bottom sediments were distinguished in the lake, taking into account its morphometry and summer oxygen concentration in water above the sediments (Fig. 1a). Zone I, characterized by the contact of sediments with anoxic waters (depth 3.0–7.3 m), had an area of 8.7 ha. The shallow zone II in which sediments are in contact with well-oxygenated epilimnetic water (0–3.0 m) had an area of 1.9 ha.

Additionally, sediments from the surface layer (ca. 10 cm) were sampled at the same two stations for the analysis of total phosphorus (TP) and phosphorus fraction content. These samples were taken at intervals of 1–2 months around the year. Phosphorus fractions were separated according to the fractioning protocol proposed by Psenner et al. (1988). In a volume of 1 cm^3^ of wet sediment, the following elements were analysed: loosely adsorbed (labile) phosphorus (NH_4_Cl-P)—extraction with 1 M NH_4_Cl; redox-sensitive phosphorus bound with iron (BD-P)—extraction with a mixture (1:1) of 0.11 M NaHCO_3_ and 0.11 M Na_2_S_2_O_4_; phosphorus bound to hydrated oxides of aluminium (NaOH-P) and organic matter (NaOH-NRP)—extraction with 1.0 M NaOH; and carbonate- and apatite-bound P (HCl-P)—extraction with 0.5 M HCl and the residue (Res-P), which was the difference between total P concentration and the sum of the first five fractions. Orthophosphates and TP concentration were also analysed in water lying above the sediments and in interstitial water, after centrifugation for 1 h at 3000 rpm in closed containers to prevent any oxidation of the samples. Sediment samples were also analysed for organic matter content (%) by drying to constant weight and combustion at 550 °C. Water content (%) was calculated from a difference between the wet and dry weight of the sample. Statistical calculations were made using STATISTICA version 10.0 software. To confirm the significance of differences between analysed sediment features in time and space, non-parametric tests were used, i.e. Kruskal-Wallis and Mann-Whitney *U* tests.

## Results

### Water in lake

The average value of water transparency in the still hypereutrophic Uzarzewskie Lake in 2008 was only 1.0 m, and it decreased to 0.4 m during summer, and concentration of chlorophyll-a reached 118.0 μg l^−1^ in the surface layer (Kowalczewska-Madura et al. 2015). Water blooms reduced the transparency to 0.6 m also in summer months in 2010–2011 and caused high oxygen concentration in the surface water layer, which reached up to 25.2 mg O_2_ l^−1^. Water pH rose up to 8.9 in the surface water layer, whilst at the bottom, it did not exceed 7.9. The lake was also characterized by a high concentration of TP, especially over the bottom, which reached up to 1.7 mg P l^−1^. The concentration of chlorophyll-a did not exceed 86.2 μg l^−1^ (Dondajewska et al. 2013, Kozak and Gołdyn 2014). The most abundant group of phytoplankton was cyanobacteria (especially in summer and autumn). The most important taxa were *Planktothrix agardhii*, *Aphanizomenon gracile*, *Pseudanabaena limnetica* and *Limnothrix redekei* (Kozak and Gołdyn 2014). The transparency in summer periods of years 2013–2015 remained at a similar level to the previous years. However, the average concentration of chlorophyll-a was reduced from approximately 58 μg l^−1^ in 2010–2011 to 38 μg l^−1^ in 2013 and next increased to 54 μg l^−1^ in the following years (unpublished data).

During the studies, the frequently mixing zone in the summer months (June–August) covered only surface water layer up to the depth of 1 m; thus, oxygen concentrations in this part of the lake exceeded 10 mg O_2_ l^−1^. Water layer from the depth of 2 m up to the bottom was characterized by oxygen content below 1 mg O_2_ l^−1^; hence, oxygen depletion near the bottom was very clear. Water mixing covered the entire water column in autumn, contributing to good oxygen conditions from the surface to the bottom. During winter months, the oxygen content variability depended on the ice cover; i.e. its presence caused lowering of oxygen concentration with depth, whilst its absence promoted high content throughout the entire water column. Distinct decrease of oxygen content from the surface to the bottom was observed already from spring, as the mixing period was very short. Usually in April–May, the oxygen concentration was below 2 mg O_2_ l^−1^ from the depth of 3–4 m to the bottom.

### Bottom sediments

The mean water content in the sediments from the deepest place in the lake reached 86.3 ± 0.683%, whilst in the sediments from the littoral zone, it reached 75.8 ± 9.61%. Dry matter clearly indicated higher values in the case of sediments from the littoral zone, on average 242 ± 96.16 g kg^−1^ wet weight (WW), against the sediments from the profundal zone, on average 137 ± 6.82 g kg^−1^ WW.

**Fig. 2:**
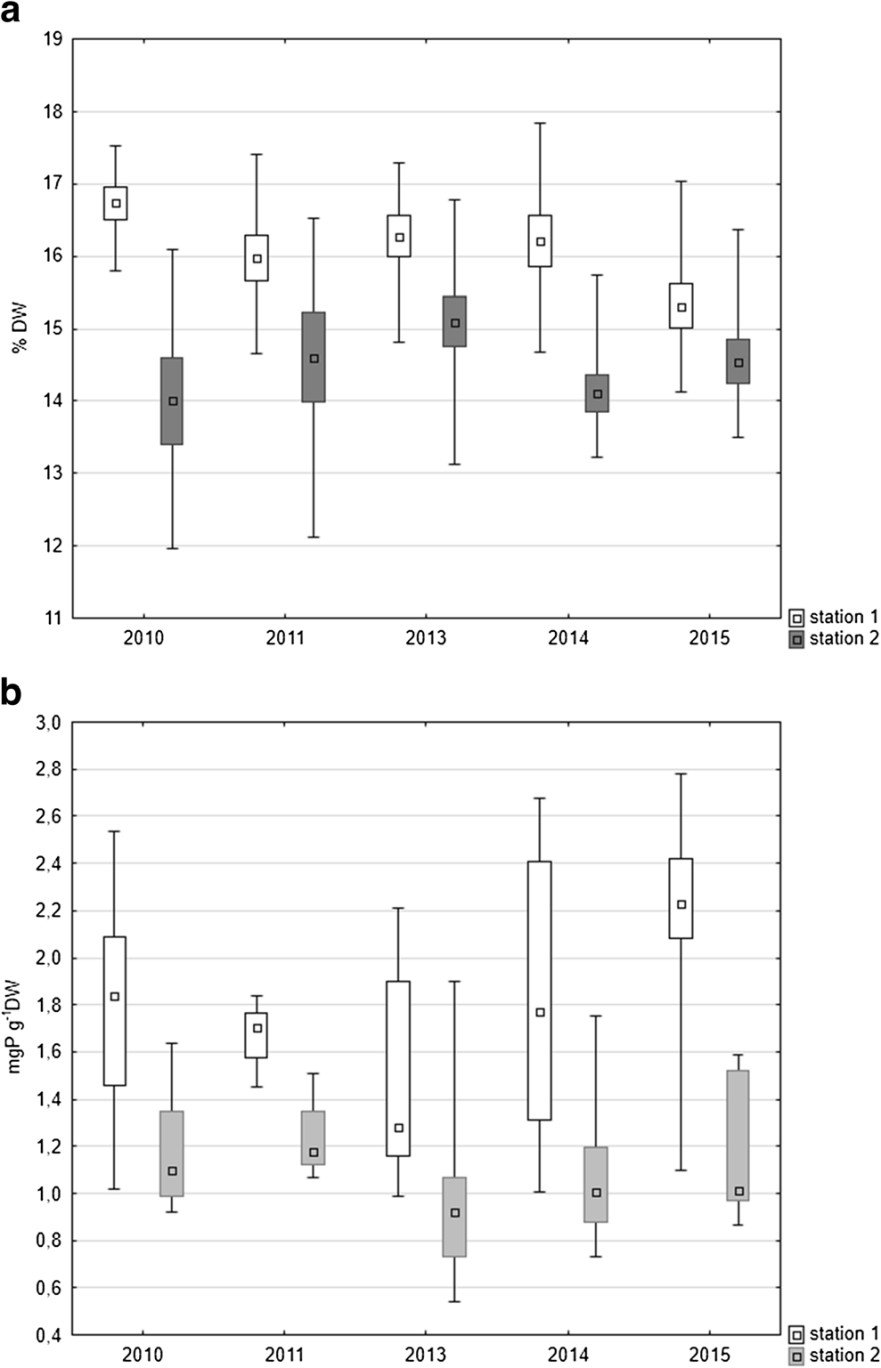
Median organic matter (**a**) and total phosphorus (**b**) content in bottom sediments of Uzarzewskie Lake in the years 2010–2011 and 2013–2015 (*box* median ± 25–75 percentile, *whiskers* minimum and maximum)

The content of organic matter indicated higher values in most cases at station 1, where they varied between 14.1 and 17.9% (median 16.29%), whilst at station 2, the variation was between 11.9 and 16.8% (median 14.43%). Higher variability of this parameter in different seasons of the year was indicated in the littoral zone of the lake (station 2) (Fig. 2a).

The mean content of organic matter in the bottom sediments of the profundal zone decreased in the following years of the research, whilst in the littoral zone, it slightly oscillated (Fig. 2a). Statistically significant differences were noted only at station 1. The results of Kruskal-Wallis non-parametric test for station 1 were *H* = 9.776, *N* = 45 and *p* = 0.044, whilst for station 2 *H* = 4.317, *N* = 45 and *p* = 0.365.

The amount of TP in the bottom sediments of the lake was higher in the profundal zone than in the littoral zone. The maximum value 2.78 mg P g^−1^ dry weight (DW) was noted in the deepest place of the lake in autumn 2015, whilst in the littoral zone, the maximum was only 1.90 mg P g^−1^ DW in autumn 2013. The minimal values, 0.99 mg P g^−1^ DW at station 1 and 0.54 mg P g^−1^ DW at station 2, were noted in June 2013. Median content of TP in the profundal sediments was 1.77 mg P g^−1^ DW, whilst in the littoral, it was 1.067 mg P g^−1^ DW. Median values indicated an upward trend in the profundal zone in the last 3 years of the study, whilst slight fluctuations were reported in the littoral zone (Fig. 2b). Similarly as for organic matter, statistically significant differences for phosphorus content in sediments were found only at station 1. The results of Kruskal-Wallis non-parametric test for station 1 were *H* = 12.431, *N* = 45 and *p* = 0.014, whilst for station 2 *H* = 3.633, *N* = 45 and *p* = 0.457.

Mann-Whitney *U* non-parametric test indicated that both in cases of organic matter and phosphorus content the differences between the analysed stations were statistically significant amounting for TP *z* = 6.358 and *p* = 0.000, for OM *z* = 5.539 and *p* = 0.000).

**Fig. 3:**
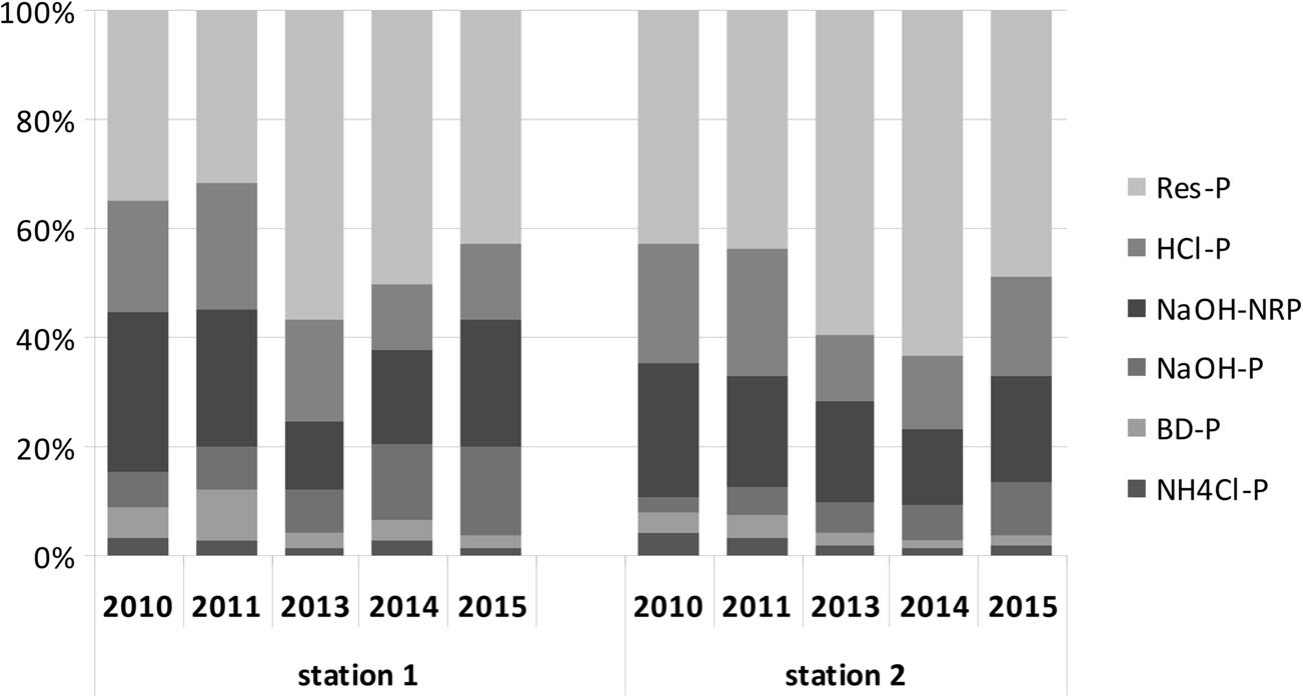
Mean of percentage share of extractable fractions of TP in bottom sediments of Uzarzewskie Lake in the years 2010–2011 and 2013–2015

The highest contribution to TP in both stations was found for the fraction Res-P characterizing phosphorus that was practically biologically unavailable, occurring in the form of insoluble compounds. Its mean contribution reached 57% at station 1 and 63% at station 2. Minor participation was observed for fractions of the highest biological availability (NH_4_Cl-P, BD-P and NaOH-P), which reached on average 6.8% for sediments from the deepest part of the lake and 4.5% for sediments from the littoral zone. When comparing the mean content of the particular fractions in the following years of the study, it was found that at both research stations, the content of the fraction characterizing loosely absorbed (labile) phosphorus on the surface of the sediment particles (NH_4_Cl-P) and the fraction of phosphorus bound with Fe (BD-P) decreased, whilst the content of the fraction bound with organic matter (NaOH-NRP) first decreased and in the following years increased. The Res-P fraction showed slightly larger fluctuations in the subsequent years of research (Fig. 3).

### Pore water

**Fig. 4:**
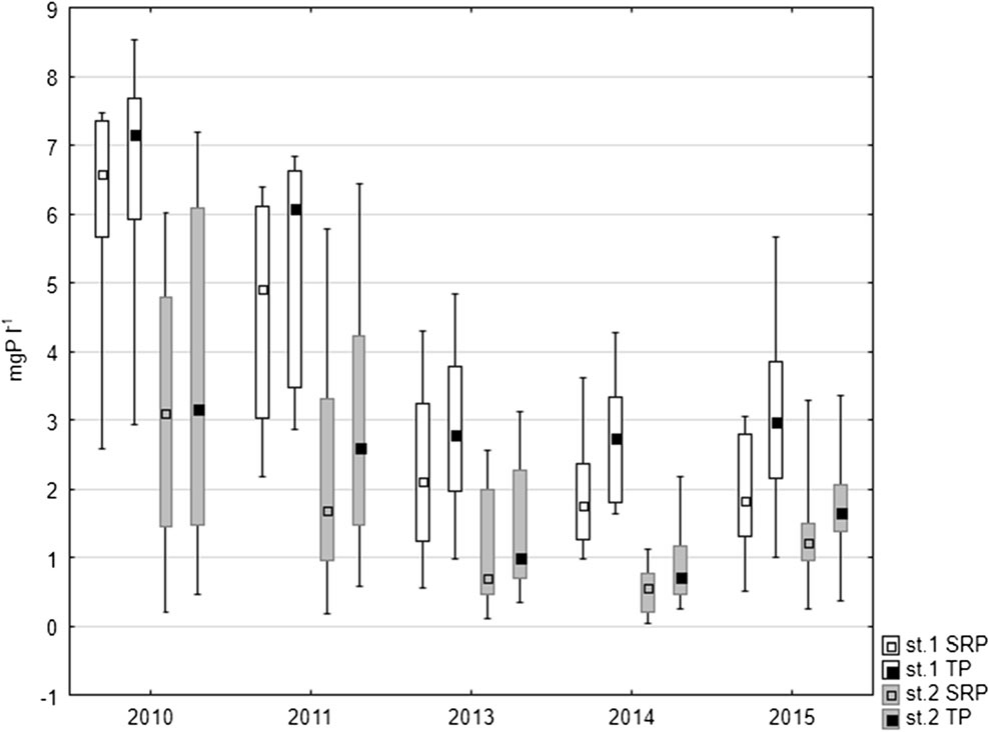
Median SRP and TP concentrations in pore waters of bottom sediments of Uzarzewskie Lake in the years 2010–2011 and 2013–2015 (*box* median ± 25–75 percentile, *whiskers* minimum and maximum)

Higher concentrations of both forms of phosphorus, i.e. soluble reactive phosphorus (SRP) and total phosphorus (TP), were noted in the interstitial waters at station 1 where they varied between 0.52 and 7.48 mg P l^−1^ in the case of SRP and between 0.98 and 8.53 mg P l^−1^ in the case of TP. At the same time, in the littoral zone, the concentrations of SRP ranged from 0.05 to 6.01 mg P l^−1^ and TP concentrations from 0.26 to 7.18 mg P l^−1^ (Fig. 4). When considering seasonal variability, the lowest values in the deepest place of the lake occurred in spring, being higher in summer and autumn. In the littoral zone, significantly higher values were also noted in summer-autumn season, whilst in the remaining seasons, they did not exceed 3 mg P l^−1^. The mean concentration of both forms of phosphorus in the interstitial water decreased in the following years of study at both research stations. A slight increase of SRP and TP concentrations was found only in 2015 at station 2 (Fig. 4).

Statistically significant differences were noted for phosphorus content in the interstitial waters between the analysed years at both research stations as well as between the stations. The results of Kruskal-Wallis non-parametric test between the analysed years were for SRP at station 1, *H* = 20.666, *N* = 43 and *p* = 0.004 and for TP *H* = 18.558, *N* = 43 and *p* = 0.001, whilst for SRP at station 2, *H* = 12.594, *N* = 43 and *p* = 0.013 and for TP *H* = 12.194 and *N* = 43, *p* = 0.016. The results of Mann-Whitney *U* test between analysed stations were for SRP *U* = 413, *Z* = 4.52, *p* = 0.000 and *n* = 43 and for TP *U* = 367, *Z* = 4.81, *p* = 0.000 and *n* = 43.

### Over lake bottom water layer

**Fig. 5:**
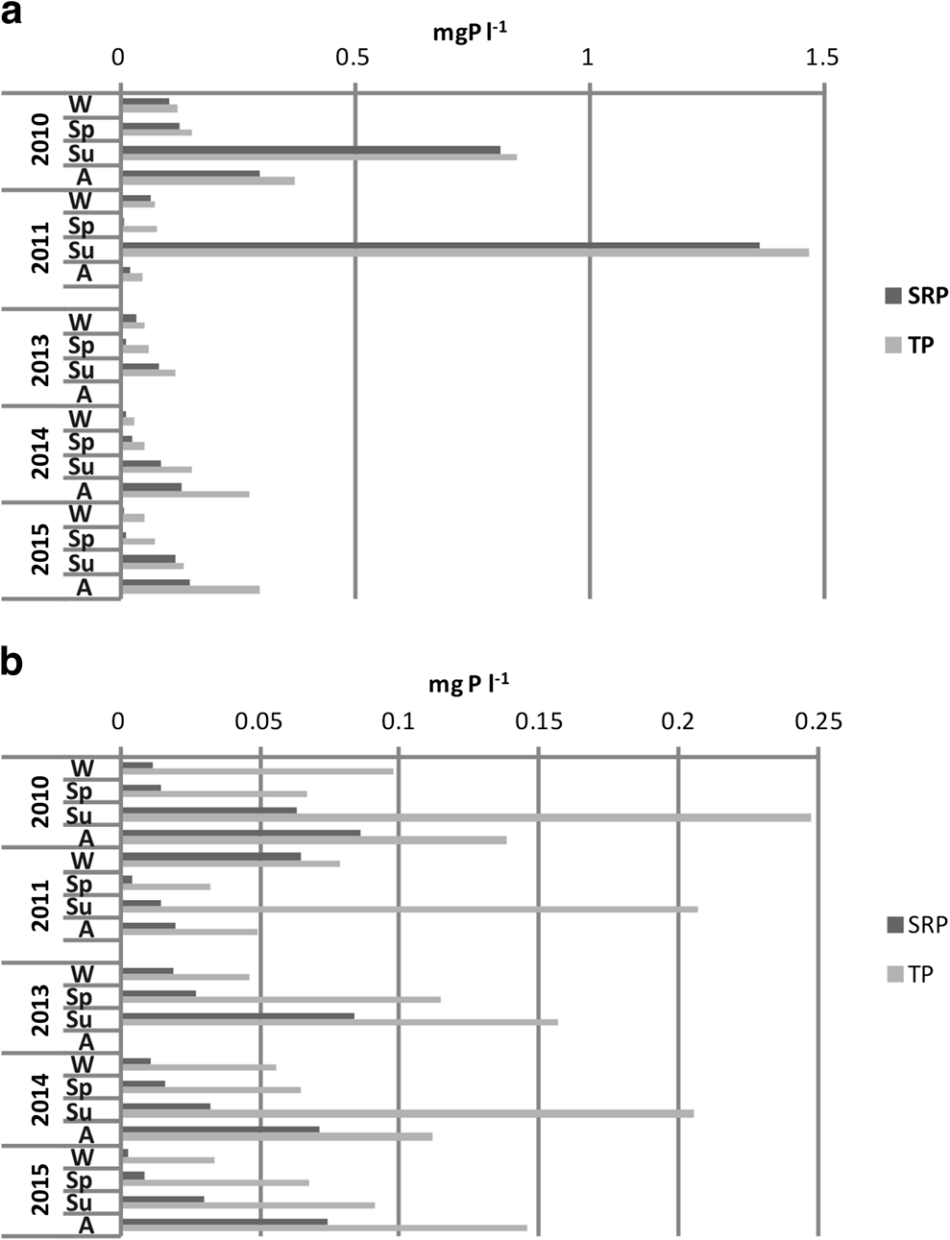
Seasonal changes of SRP and TP concentrations in over lake bottom waters of Uzarzewskie Lake in the years 2010–2011 and 2013–2015 at station 1 (profundal zone) (**a**) and station 2 (littoral zone) (**b**). *W* winter, *Sp* spring, *Su* summer, *A* autumn

Concentrations of dissolved phosphates and TP in the over-bottom water layer also indicated a clear seasonal variability in the research period. At station 1 in the profundal zone, the highest values were noted in summer periods of both years 2010–2011 and of 2013. In the years 2014–2015, increased values of these parameters were also observed in autumn, when they exceeded the levels of summer (Fig. 5a).

The maximum concentration of SRP in 2010 reached 0.81 mg P l^−1^ and TP 0.85 mg P l^−1^, whilst they were higher in 2011 and reached 1.36 and 1.47 mg P l^−1^, respectively. A clear decrease in these forms of phosphorus was visible in the years 2013–2015. They ranged up to 0.15 mg P l^−1^ for the SRP and 0.30 mg P l^−1^ for TP in autumn 2015. Lower concentrations of both forms of phosphorus were noted in other seasons (Fig. 5a).

**Fig. 6:**
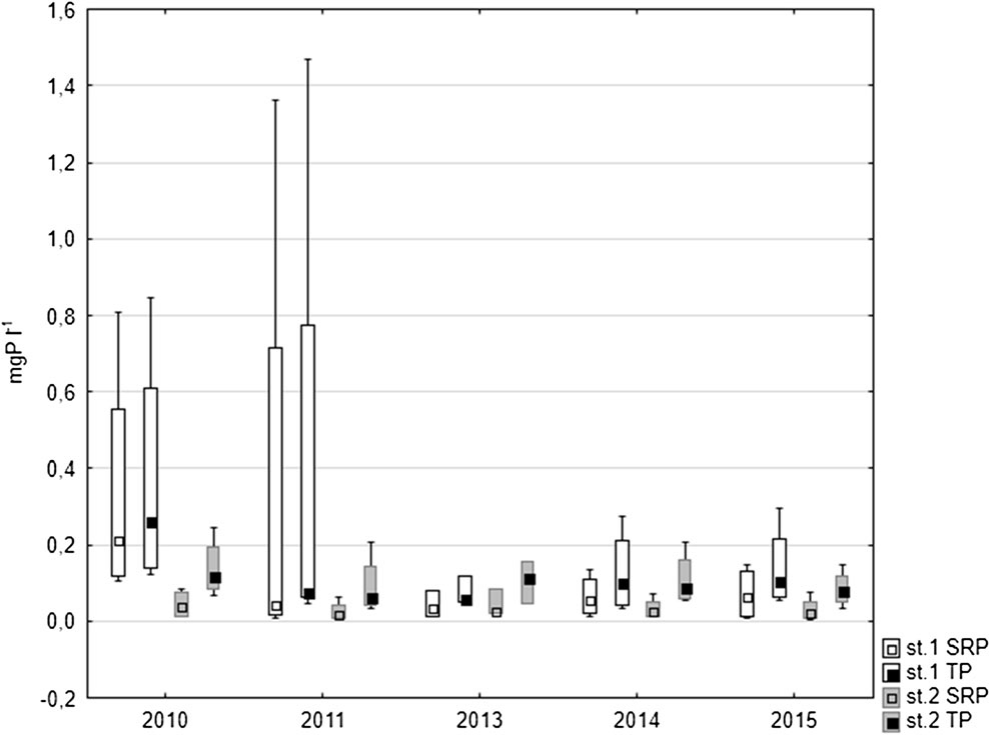
Median SRP and TP concentrations in over lake bottom waters of Uzarzewskie Lake in the years 2010–2011 and 2013–2015 (*box* median ± 25–75 percentile, *whiskers* minimum and maximum)

At station 2, situated in the littoral zone, these values, especially in years 2010–2011, were significantly lower than at station 1. In succeeding years of the study, the concentrations of both forms of phosphorus in the over-bottom waters of the littoral were only slightly lower than those found in the profundal. Their maximum reached up to 0.09 mg P l^−1^ for SRP and up to 0.25 mg P l^−1^ for TP. The highest values of TP were observed during summer, and only in the last year of the study, they were higher in autumn (Fig. 5b). The median values decreased in the profundal zone (both SRP and TP), especially in the years 2013–2015. Slightly higher values were found in the littoral zone in 2010, whilst in the following years, they were similar to each other (Fig. 6).

### Phosphorus release or accumulation in bottom sediments of Uzarzewskie Lake

**Fig. 7:**
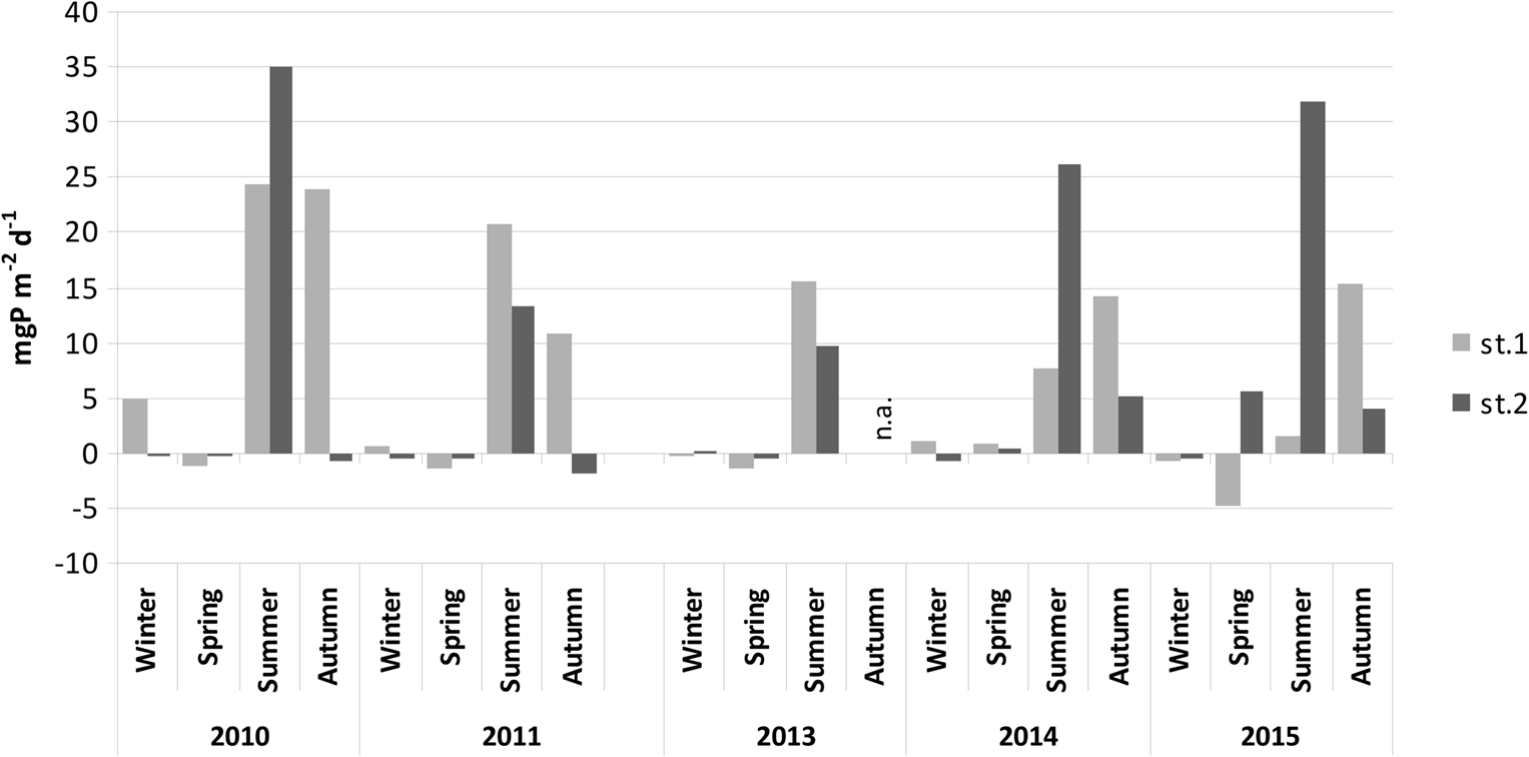
Seasonal changes of domination of phosphorus release (*positive values*) or accumulation in bottom sediments (*negative values*) of Uzarzewskie Lake at both stations in the years 2010–2011 and 2013–2015 (*n.a.* not analysed)

Ex situ experiments on phosphorus internal loading from the bottom sediments revealed a clear variability in the process, both in time and in different parts of the lake. At the station located in the profundal zone, a predominance of phosphorus release over its accumulation was noted in the greater part of the research period. The highest values were found in summer periods of the first 2 years of the study (Fig. 7). They reached up to 24.5 mg P m^−2^ day^−1^ in 2010 and to 20.8 mg P m^−2^ day^−1^ in 2011. In subsequent years, these values were reduced gradually to 1.5 mg P m^−2^ day^−1^ in 2015. Relatively high values were also noted in autumn especially in 2010, when phosphorus release from bottom sediments reached 23.7 mg P m^−2^ day^−1^, whilst it was twice as low in 2011. Phosphorus release in autumn 2013 was not analysed, but in subsequent years, it was at a similar level, close to 15.0 mg P m^−2^ day^−1^. Phosphorus release also predominated over its accumulation in winter, but the difference was minor, reaching 4.92 mg P m^−2^ day^−1^ in 2010 and not exceeding 1.0 mg P m^−2^ day^−1^ in subsequent years. The opposite process predominated in spring both in 2010 and 2011 and in winter and spring in 2013 and 2015. The accumulation of phosphorus in the bottom sediments increased, reaching 4.8 mg P m^−2^ day^−1^ in spring 2015 (Fig. 7).

Phosphorus release from the bottom sediments of the littoral zone of Uzarzewskie Lake in years 2010–2011 and 2013 was observed only in summer. It reached 35.0 mg P m^−2^ day^−1^ in 2010, and it significantly dropped in 2011 and in 2013 (Fig. 7). The released amount in 2010 and 2014–2015 was even higher than noted in the profundal zone. In the years 2014–2015, an advance of this process was also observed in spring and autumn. Its accumulation in the littoral zone predominated over its release in the remaining periods in 2010–2011 and varied between 0.18 and 1.78 mg P m^−2^ day^−1^. In the following years, the accumulation of phosphorus in the bottom sediments was found only in spring of 2013 (0.61 mg P m^−2^ day^−1^) and in winter 2014 and 2015 (up to 0.59 mg P m^−2^ day^−1^) (Fig. 7).

**Fig. 8:**
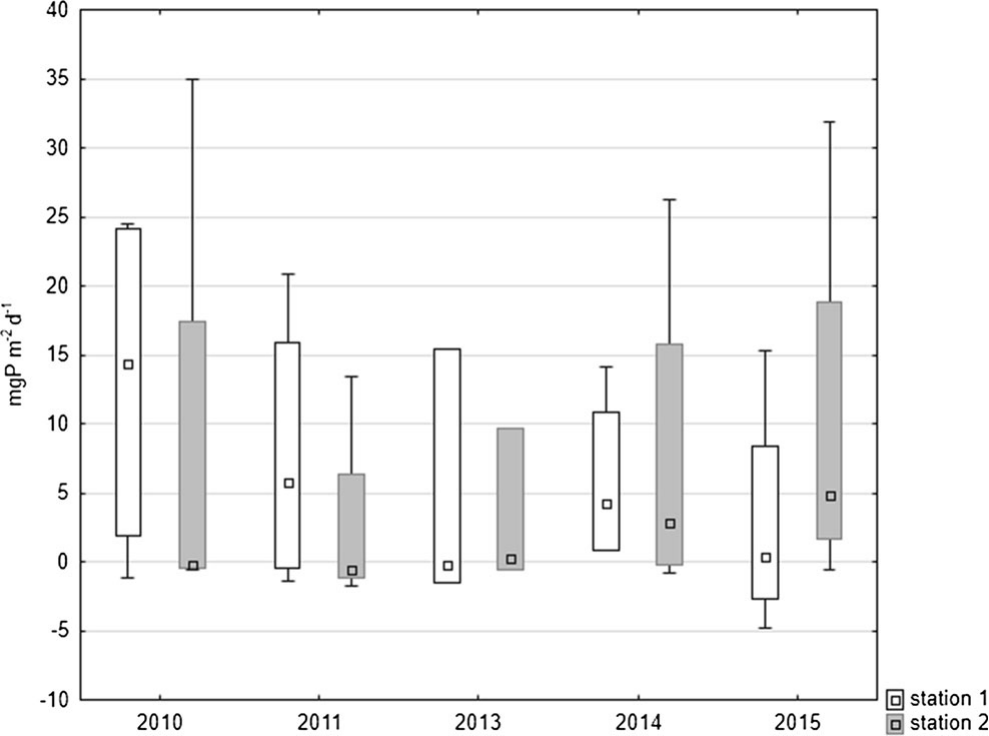
Comparison of medians of phosphorus release from bottom sediments at both stations in Uzarzewskie Lake in the years 2010–2011 and 2013–2015 (*box* median ± 25–75 percentile, *whiskers* minimum and maximum)

Lower median values of internal loading occurred in the littoral zone than in the deepest part of the lake in 2010–2011 and 2013. At both stations, these values dropped in 2011 and 2013 against the values from 2010 (Fig. 8). The internal loading of phosphorus increased in the following years in the littoral zone up to 4.835 mg P m^−2^ day^−1^ in the last year of the study. The median annual value of internal P loading in the profundal zone displayed a decreasing tendency in almost all the years of the study down to 0.386 mg P m^−2^ day^−1^ (Fig. 8). As a result of this, the median of the whole period of research was only slightly higher in the profundal zone reaching 1.486 mg P m^−2^ day^−1^ than in the littoral zone 0.29 mg P m^−2^ day^−1^.

**Fig. 9:**
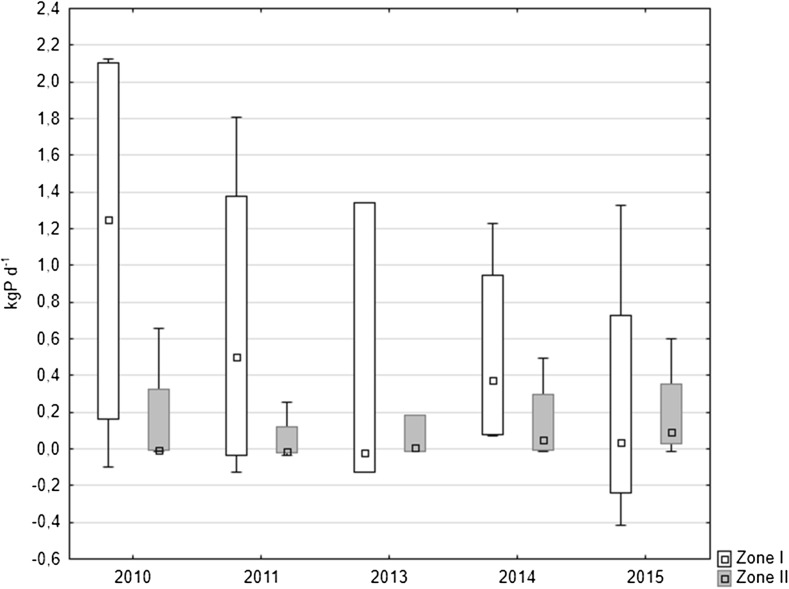
Total phosphorus loading from the both zones of Uzarzewskie Lake in the years 2010–2011 and 2013–2015 (*box* median ± 25–75 percentile, *whiskers* minimum and maximum)

The internal loading of phosphorus was also calculated for the whole area of two zones in the lake, which differed in water depth and oxygenation, especially in summer. Much more important in supplying the lake with phosphorus was the profundal zone, in which a higher P release was observed. This area was larger than the littoral zone (82% of the lake surface). Internal phosphorus loading in the profundal zone was reduced in the following years from median value 1.25 kg P day^−1^ in 2010 to 0.034 kg P day^−1^ in 2015 (i.e. by around 78%) (Fig. 9). In the littoral zone, a decrease of the phosphorus internal loading to 0.005 kg P day^−1^ was observed in 2013. There was an increase of median to 0.053 and 0.091 kg P day^−1^ in the period 2014–2015, respectively (Fig. 9).

Considering the annual phosphorus loads released from two zones of the lake, it can be concluded that in the whole period of the study, it was higher in the profundal zone (zone I) than in the littoral (zone II). In zone I, it decreased over the years of the research. The total load of phosphorus from bottom sediments of the two zones of the lake decreased more markedly in the last year of the study, approximately 66% when compared to 2010 (Table 4).

**Table 4:** Annual changes of phosphorus release from bottom sediments of Uzarzewskie Lake and total phosphorus loading from the both zones of the lake

Year	Mean P release (SD) (mg P m^−2^ day^−1^)		Mean P loading from the zones (kg P a^−1^)		Total P load (kg P a^−1^)
Station/Zone	Station 1	Station 2	Zone I	Zone II	Zone I + Zone II
2010	13.02 (13.0)	8.46 (17.7)	413.2	58.4	471.6
2011	7.71 (10.3)	2.61 (7.2)	244.9	18.1	263.0
2013	4.58 (9.4)	3.12 (5.1)	145.3	21.5	166.8
2014	5.88 (6.4)	7.75 (12.6)	186.5	53.7	240.2
2015	2.81 (8.7)	10.24 (14.5)	89.1	70.5	159.6

*SD* standard deviation

## Discussion

Highly eutrophicated lakes, with intense water blooms caused by phytoplankton, need protection and/or restoration treatments in order to improve their water quality. In the case of Uzarzewskie Lake, a chemical method of restoration was used in 2006–2007, namely phosphorus inactivation with the use of iron sulphate (Kowalczewska-Madura et al. 2010). Another method was used from 2008 when the cold and nitrate-rich water of seepage springs was directed towards the hypolimnion to raise the redox potential of the bottom sediments (Gołdyn et al. 2014).

When analysing the variability of phosphorus release from the bottom sediments of Uzarzewskie Lake, significantly higher values were found in the profundal zone compared to the littoral zone. Such differences were found in this lake in previous years 2005–2008 (Kowalczewska-Madura et al. 2008, 2010, 2015). The higher values of phosphorus release in the profundal zone were connected with oxygen depletion in the over-bottom waters, which occurred especially in summer and early autumn. Good aerobic conditions in the lake were found only during early spring and late autumn water mixing. Because of the ice cover in winter oxygen concentration in the over-bottom water layer fell but did not decrease below 2.8 mg O_2_ l^−1^ (Dondajewska et al. 2013), it was enough that reductive processes did not lead to the release of phosphorus from the sediments. A similar pattern of oxygen distribution in the vertical profile of the lake was already found previously (Gołdyn et al. 2008, Kowalczewska-Madura et al. 2010). Anaerobic conditions are one of the most important causes of a significant phosphorus release from bottom sediments (Ishikawa and Nishimura 1989). Under aerobic conditions, iron is in the form of insoluble compounds and adsorbs phosphorus. Under anaerobic conditions, iron is reduced and its compounds are soluble releasing phosphorus to the water column (Søndergaard et al. 2002). The presence of hydrogen sulphide indicates strong reducing conditions, which causes the reduction of iron and the formation of iron sulphide (Kleeberg et al. 2013).

The colour of sediments in Uzarzewskie Lake was black in the first years of restoration, indicating the reduced status and presence of FeS (Søndergaard et al. 2002). Such conditions existed still in 2010–2011 in the profundal zone in summer, which caused strong P release. High phosphorus release from bottom sediments was also noted in autumn, despite the oxygenation of the sediment-water interface during autumn turnover. This release in the lake can be additionally stimulated by a more rapid temperature decrease in water than in sediment (Golosov and Ignatieva 1999). Due to the thermal convection of interstitial water, phosphorus can be transported from the bottom sediments to the water column.

Prolonged and intensive release of phosphorus in summer and autumn contributed to the lowest content of TP in bottom sediments at that time. The reverse process of phosphorus accumulation in the bottom sediments predominated during spring turnover period, which was a result of both water oxygenation and low sediment temperature. Due to this process, the highest content of TP in the sediments was noted in spring. In comparison to the results from the previous years (2005–2008), it was found that the lowest values were recorded in 2006, when the highest internal loading was observed (Kowalczewska-Madura et al. 2008, 2010).

The process of phosphorus accumulation predominated in the littoral zone over its release to the water column in the years 2010–2011 and 2013. Only in summer, as a result of a significant increase in water temperature and a decrease in oxygen concentration at the sediment-water interface during calm weather did phosphorus release from the bottom sediments predominate. Such a high intensity of the process, noted mainly in summer 2010, was caused by oxygen depletion in the lake from the depth of 2 m (0.38 mg O_2_ l^−1^) and the high temperature of the over-bottom water layer in the littoral zone, reaching 23 °C (unpublished data). Good oxygenation of the epilimnion waters in the remaining seasons of the year led to P accumulation in the bottom sediments of this zone. In 2014–2015, a predominance of the release of phosphorus from the sediments on its accumulation in the littoral zone was also observed in autumn and partially in spring in addition to summer period. The results therefore prove that the restoration processes conducted in subsequent years effectively blocked the release of phosphorus from sediments in the profundal zone but not in the littoral zone.

This relationship between the phosphorus accumulation process and its release from bottom sediments and the oxygen conditions and temperature of the sediments from the littoral zone was confirmed by statistically significant correlations. The process of internal loading was positively correlated with temperature (*r* = 0.849, *p* = 0.00) and negatively correlated with the dissolved oxygen concentration (*r* = −0.66, *p* = 0.002) and conductivity (*r* = −0.54, *p* = 0.017). Such a correlation was not found for the bottom sediments from the profundal zone.

The process of internal loading was also correlated with the concentration of TP in the over-bottom water layer. This was confirmed by a positive statistically significant correlation for both research stations (*r* = 0.689 at station 1 and *r* = 0.690 at station 2, *p* < 0.05). In the case of these two parameters, a similar seasonal variability was found. Phosphorus released from the sediments was retained in the hypolimnetic water layer during thermal stratification (station 1) and in the over-bottom water layer in the littoral zone in periods of calm weather (station 2).

The analysis of the variability of SRP and TP concentrations in the interstitial water of Uzarzewskie Lake showed that higher values were noted in summer and autumn periods at the station located in the profundal zone, as phosphorus released by the mineralization of organic matter in the sediments was enriching the interstitial water first; this was also found by Head et al. (1999). A similar seasonal variability was recorded in the littoral zone. It was confirmed by statistically significant correlations between the intensity of the internal loading process and the SRP and TP concentrations in the interstitial waters. We found a positive correlation for both stations: in the profundal zone (*r* = 0.470, *p* = 0.042) and in the littoral zone (*r* = 0.610, *p* = 0.005). Phosphorus concentrations in pore waters and above the sediments are commonly regarded as indices of the intensity of its transport across the sediment-water interface (Kentzer 2001; Komatsu et al. 2006). A similar dependence between these parameters was also noted in Lake Swarzędzkie (Kowalczewska-Madura and Gołdyn 2012). The concentration of phosphorus in the pore water decreased in the first years of the study, which resulted in a reduction of the internal loading. As a result of the restoration process, concentration of phosphorus in sediments of the profundal zone visibly increased in the years 2013–2015, although its concentration in pore water was at a similar level. Moreover, phosphorus release was at a similar level in these zones. This was undoubtedly a result of the increase in the redox potential of sediments, manifested by a lack of hydrogen sulphide and an increase in the sorption complex of sediments in relation to phosphorus.

**Fig. 10:**
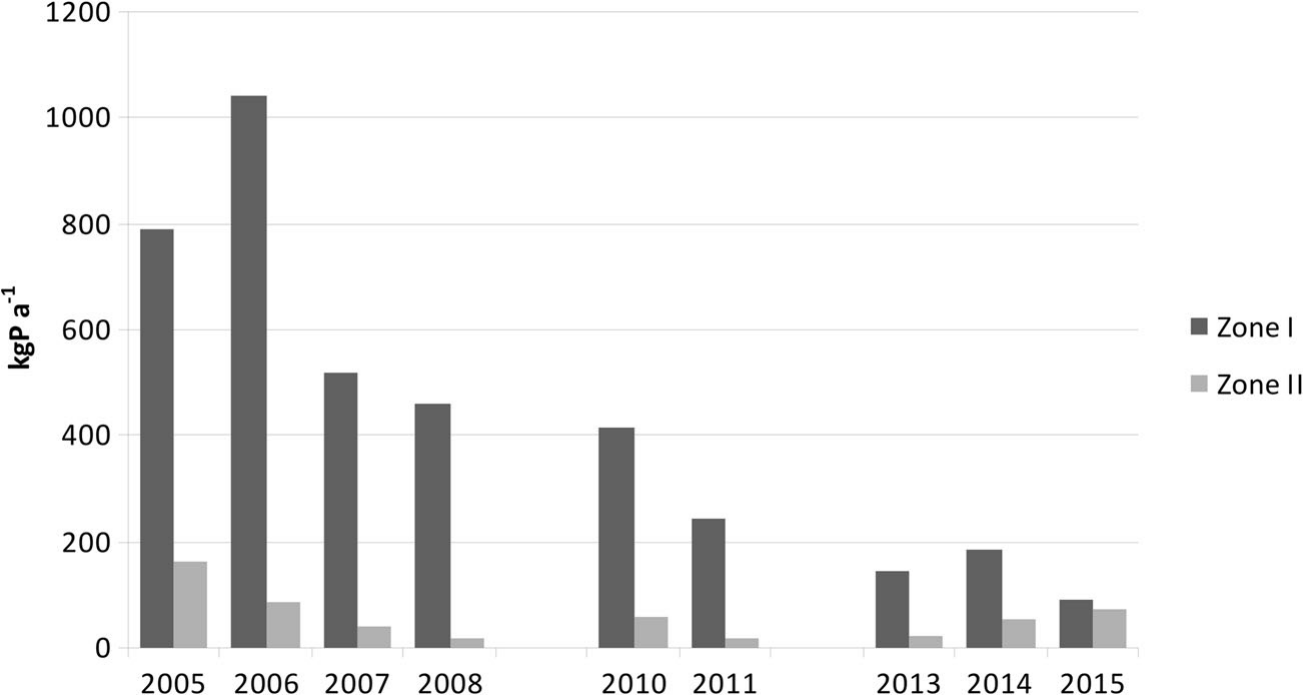
Annual internal loading of phosphorus in Uzarzewskie Lake in the periods 2005–2011 and 2013–2015 (according to Kowalczewska-Madura et al. 2008, 2010, 2015 and current data)

Whilst comparing the results of the present research with the data from previous studies (Kowalczewska-Madura et al. 2008, 2010, 2015), concerning the period before (2005), during the lake restoration with PIX (2006–2007) and beginning of hypolimnion supply with spring waters (2008–2009), it was found that the annual phosphorus loading from bottom sediments was clearly decreasing. The highest annual load from the profundal zone 1040.8 kg P a^−1^ was noted in 2006 whilst the lowest, 89.1 kg P a^−1^ was recorded in 2015 (Fig. 10). In the littoral zone, it was significantly lower, decreasing from its maximum of 162.4 kg P a^−1^ in 2005 to the minimum 17.8 kg P a^−1^ in 2008 due to iron treatment. In the following years of research, it increased to 70.5 kg P a^−1^ in 2015 (Fig. 10), as the new restoration strategy of supplying spring water to the profundal zone did not include the littoral zone.

The obtained results clearly illustrate the role of the release of phosphorus from the two different zones of Uzarzewskie Lake. Despite its increase in the littoral zone in recent years of the research, the phosphorus load in this zone is still smaller than in the profundal. Problematic is, however, that phosphorus released from the sediment in the littoral zone passes directly to the trophogenic zone, causing water blooms, especially in summer. This would seem to provide clear proof as to why no significant improvement in water quality was observed in Uzarzewskie Lake (Kowalczewska-Madura et al. 2015).

Whilst summing up the load from both zones in Uzarzewskie Lake, it was found that a gradual decrease was also occurring over successive years. Its highest value was noted in 2006; by 2011, this decreased by 77%, and considering the period prior to the restoration treatment, there was a reduction of 72%. By 2015, this decrease was 85.9 and 83.2%, respectively.

Phosphorus content in bottom sediments belonging to various fractions was altered over time under the influence of restoration. Despite the iron treatment in 2007, the P fraction associated with iron (DB-P) was very low in the profundal zone, averaging 0.07 mg P g^−1^ DW. This was related to the strong deficits of oxygen and very low redox potential that promotes the release of P from the bottom sediments (Sobczyński and Joniak 2009, Kowalczewska-Madura et al. 2010). Increasing this potential in 2008 by providing spring waters containing nitrates to the bottom of the lake, whilst it did not increase the oxygen concentration, resulted in an increase in redox potential and the persistence of oxidized iron compounds that inactivate phosphorus in bottom sediments (Gołdyn et al. 2014). In 2008, the fraction of BD-P ranged between 4.94 and 33.17% of TP in the sediment, representing an average of 0.37 mg P g^−1^ DW (Kowalczewska-Madura et al. 2015). In the studied periods 2010–2011 and 2013–2015, this fraction was much lower (Fig. 4), which may be associated with the storage of a new layer of deposits, isolating compounds of iron stored in 2006–2007, during iron treatment. Hence, organic bound P is a more important fraction now, reaching 0.42–0.56 mg P g^−1^ DW (Fig. 4), whilst in 2008, it was 0.34 mg P g^−1^ DW (Kowalczewska-Madura et al. 2015).

The phosphorus fraction which dominated in the bottom sediments of the lake is phosphorus permanently bound in non-biodegradable compounds (Res-P). A similar contribution of this fraction was found in Swarzędzkie Lake, which is situated in the same river course (Kowalczewska-Madura et al. 2005) as well as in many other lakes (Fytianos and Kotzakioti 2005, Brzozowska et al. 2007). This fraction is practically useless in the P release from bottom sediments (Kentzer 2001). The Res-P fraction characterized by the lowest solubility and bioavailability is permanently accumulated in bottom sediments. It is favourable for water quality because it limits the influence of bottom sediments as a source of internal phosphorus loading. We found a negative correlation between Res-P fractions and the concentration of TP in the interstitial water at both sampling stations (*r* = −0.45 and *r* = −0.49, *p* < 0.05, respectively), which suggests that this fraction does not participate in phosphorus release either to pore water or to near bottom water.

Unfortunately, this form of phosphorus showed a small downward trend in the years 2010–2011 and 2013–2015. It was, on average, as much as 45.9% in 2008 in the profundal sediments (Kowalczewska-Madura et al. 2015), whilst in the studied years, it was 43.2%. This trend is fortunately compensated by the growing importance of the HCl-P fraction, which in 2008 accounted for 15.0% and in the period 2010–2015 17.8%. Admittedly, the mean contribution of three bioavailable fractions jointly (NH_4_Cl-P, BD-P and NaOH-P) to the TP was low. This may be related to their release into the water column (Ting and Appan 1996; Kaiserli et al. 2002; Pardo et al. 2003). The mobility of the fraction may be supported by an index of concentration variability (maximum/minimum) (Kentzer 2001). In the case of profundal and littoral sediments in Uzarzewskie Lake, the ratio was highest for fraction BD-P. Mean contribution of the fraction was low, which confirmed their release from the sediments. Moreover, a statistically significant positive correlation between the content of fraction BD-P in the profundal sediments and the concentration of SRP in the interstitial water (*r* = 0.33, *p* = 0.01) may indicate the role of this fraction in phosphorus release from the sediments to the pore water and next to the water column.

Steady decreasing trend, which was observed in the internal loading of phosphorus, was connected with the restoration measures expressed by the gradual sustainable rebuilding of the entire ecosystem. It consisted of decrease of primary production and organic matter sedimentation, reducing the amount of organic matter readily decomposed by microbes (Katsev and Dittrich 2013).

Nevertheless, the internal P loading in Uzarzewskie Lake is still at least three times higher if compared to less eutrophicated lakes (Kowalczewska-Madura et al. 2015) and causes water blooms and a high concentration of chlorophyll-a. Therefore, it is necessary to reduce the external loading of this lake. The most important measure, i.e. the diversion of treated sewage from the village of Uzarzewo, was already accomplished in 2015. A further important measure would also be to reduce the internal phosphorus loading from sediment in the littoral zone. This could be achieved by inactivation of phosphorus in the sediments of this zone, e.g. by a reprise of iron treatment or Phoslock dosage.

## Conclusions

The experimental research on the intensity of internal loading from the bottom sediments of Uzarzewskie Lake showed a gradual downward trend over time. A comparison with previous results demonstrates that it is a long-term trend associated with the two different sustainable methods of restoration applied to this lake. Unfortunately, the content of phosphorus is still too high, which causes water blooms, so decreasing both external and internal loading from the littoral zone would be very advisable. The restoration process is usually long term and shows positive results in a longer-time perspective. This statement is especially true in case of sustainable restoration of hardly degraded water ecosystems, where small doses of chemical compounds are used, as it is in the case of Uzarzewskie Lake. The reduction of internal P loading in profundal area of this lake should be—in our opinion—recognized as a restoration success. This result was observed in multiannual research, and only this kind of approach allows noticing the reactions of ecosystem to conducted treatment. Internal loading is usually analysed in short-time seasonal aspects; hence, our long-term research covering both times prior the restoration as well as during the process itself broadens the knowledge on lake ecosystem response to the restoration process.
